# Molecular Characterization and Establishment of a Prognostic Model Based on Primary Immunodeficiency Features in Association with RNA Modifications in Triple-Negative Breast Cancer

**DOI:** 10.3390/genes14122172

**Published:** 2023-12-02

**Authors:** Hongzhuo Xia, Xi Xu, Yuxuan Guo, Xiyun Deng, Yian Wang, Shujun Fu

**Affiliations:** 1 The Key Laboratory of Model Animals and Stem Cell Biology in Hunan Province, Departments of Pathology and Pathophysiology, Hunan Normal University School of Medicine, Changsha 410013, China; xiahongzhuo@gmail.com (H.X.); xuxi6868@163.com (X.X.); 202320193547@hunnu.edu.cn (Y.G.); dengxiyunmed@hunnu.edu.cn (X.D.); 2 The Key Laboratory of Translational Cancer Stem Cell Research, Department of Pathophysiology, Hunan Normal University, Changsha 410012, China; 3 The Engineering Research Center of Reproduction and Translational Medicine of Hunan Province, Changsha 410013, China

**Keywords:** triple-negative breast cancer, primary immunodeficiency, prognosis model, RNA modification, immunotherapy

## Abstract

Triple-negative breast cancer (TNBC) is the most aggressive subtype of breast cancer. Although immunotherapy is effective for some patients, most find it difficult to benefit from it. This study aims to explore the impact of specific immune pathways and their regulated molecular mechanisms in TNBC. The gene expression data of breast cancer patients were obtained from the TCGA and METABRIC databases. Gene set variation analysis (GSVA) revealed specific upregulation or abnormal expression of immunodeficiency pathways in TNBC patients. Multi-omics data showed significant differential expression of Primary Immunodeficiency Genes (PIDGs) in TNBC patients, who are prone to genomic-level variations. Consensus clustering was used in two datasets to classify patients into two distinct molecular subtypes based on PIDGs expression patterns, with each displaying different biological features and immune landscapes. To further explore the prognostic characteristics of PIDGs-regulated molecules, we constructed a four-gene prognostic PIDG score model and a nomogram using least absolute shrinkage and selection operator (LASSO) regression analysis in combination with clinicopathological parameters. The PIDG score was closely associated with the immune therapy and drug sensitivity of TNBC patients, providing potential guidance for clinical treatment. Particularly noteworthy is the close association of this scoring with RNA modifications; patients with different scores also exhibited different mutation landscapes. This study offers new insights for the clinical treatment of TNBC and for identifying novel prognostic markers and therapeutic targets in TNBC.

## 1. Introduction

Triple-negative breast cancer (TNBC) is the most aggressive subtype, accounting for 10–20% of breast cancer cases [1]. Due to the lack of estrogen receptors (ER), progesterone receptors (PR), and human epidermal growth factor receptor 2 (HER2), TNBC has a higher recurrence rate and limited response to conventional treatments compared to other subtypes of breast cancer, making it the subtype with the worst clinical prognosis [2,3]. Therefore, exploring the set of genes with abnormal expression in TNBC compared to non-TNBC and analyzing the mechanisms behind TNBC development, as well as their relationship with patient prognosis, is crucial for clinical treatment.

Through GSVA analysis, we observed a significant increase in the abnormal expression of Primary immunodeficiency (PID) in TNBC compared to non-TNBC. This suggests a potential close association between PID and the onset or treatment of breast cancer. PID refers to congenital immune system abnormalities caused by genetic mutations, which are characterized by recurrent and life-threatening infections, autoimmune diseases, and cancer, posing significant challenges for diagnosis and treatment [4]. Many PIDs are associated with malignant tumors, each with distinct mechanisms underlying cancer development [5,6]. However, their specific mechanisms in TNBC remain unclear. The observed higher incidence of cancer in individuals with PIDs underscores the role of the immune system in controlling malignant tumor progression [7]. Furthermore, it is worth exploring whether treatment targeting PID can be combined with existing treatment methods to achieve enhanced therapeutic efficacy. Hence, it is crucial to explore personalized immunotherapies targeting PID-associated pathways in TNBC. In the presence of PID, immunotherapy offers promising treatment modalities for clinicians combating malignant tumors.

Currently, treatment options for TNBC remain limited, with neoadjuvant chemotherapy being one of the main treatment modalities [8]. Nonetheless, immunotherapy is considered to have significant potential in TNBC treatment [9,10,11]. The most successful immunotherapy currently involves immune checkpoint inhibitors (ICIs) [12], aimed at enhancing the cytotoxicity and proliferation capacity of tumor-infiltrating lymphocytes (TIL), although it has not yielded favorable results in TNBC [13,14]. Therefore, finding effective treatment drugs and targets remains an urgent and crucial challenge in the clinical practice of TNBC.

This study systematically analyzes the expression of Primary Immunodeficiency Genes (PIDGs) and their impact on the development, prognosis, tumor microenvironment, and treatment response of TNBC patients. Using the cancer genome atlas program (TCGA) and the molecular taxonomy of breast cancer international consortium (METABRIC) databases, two distinct PID subgroups were identified in TNBC, and their molecular characteristics and immune cell infiltration were studied. By constructing the PIDG score, the relationship between the PIDG score and RNA modification-related genes was examined, predicting the clinical prognosis, immune therapy effectiveness, and clinical chemotherapeutic drug efficacy for TNBC patients. We hope this study will potentially contribute to clinical treatment and possibly provide new directions in TNBC therapy.

## 2. Methods

### 2.1. Data Collection

Gene expression data, clinical information, copy number variations, and mutation data for 1113 breast cancer (BRCA) tumor samples and 113 adjacent non-cancer samples were downloaded from the the cancer genome atlas program (TCGA) database (https://portal.gdc.cancer.gov) with a retrieval date of 17 August 2023. BRCA patients with clinical immunohistochemistry information indicating “breast_carcinoma_estrogen_receptor_status”, “breast_carcinoma_progesterone_receptor_status”, and “lab_proc_her2_neu_immunohistochemistry_receptor_status” as “Negative” were selected for TNBC samples. Ultimately, 116 TNBC samples were screened and identified, with 115 having survival information.

Gene expression profiles and clinical data for 2509 breast cancer samples were obtained from the molecular taxonomy of breast cancer international consortium (METABRIC) database through the cBioPortal (https://www.cbioportal.org, accessed on 17 August 2023) with a retrieval date of 26 September 2023 [15]. A total of 320 patients with clinical information “ER_STATUS”, “HER2_STATUS”, and “PR_STATUS” as “Negative” were selected for TNBC samples. These samples ultimately had complete survival information.

### 2.2. Collection of m1A/m5C/m6A/m7G-Related Modification Genes

The writer, reader, and eraser genes of m6A/m1A/m5C were obtained from the literature [16]. The relevant genes for m7G were obtained from the literature [17], and duplicate genes were removed (Table 1).

### 2.3. Functional Enrichment Analysis

We utilized the kyoto encyclopedia of genes and genomes (KEGG) gene set (c2.cp.kegg.v7.4) [18] and performed GSVA on breast cancer samples.

### 2.4. Analysis of Gene Variations

The mutation landscape of genes was analyzed using the “maftools” R package (version 4.3.1) [19], which included the frequency of copy number variations (CNVs) and their locations on chromosomes.

### 2.5. Identifying Hub Genes in PPI

The protein–protein interaction networks (PPI) data for these genes was obtained from the STRING database “https://string-db.org (accessed on 7 October 2023)” with a confidence level > 0.400. Using Cytoscape v3.10.1, hub genes were filtered based on their degree of interaction.

### 2.6. Consensus Clustering Analysis

We utilized the “ConsensusClusterPlus” package for consensus clustering analysis. We conducted 50 iterations with an 80% resampling rate, employing the k-means clustering algorithm to ensure the stability of classification. Principal component analysis (PCA) was performed to explore the distribution of samples between different clusters.

### 2.7. Analysis of the Biological Features of PID Subtypes

Gene Set Enrichment Analysis (GSEA) was used to investigate the functions of different subgroups in TNBC. Single sample Gene Set Enrichment Analysis (ssGSEA) was employed to evaluate the immune infiltration status in TNBC patients and the difference in immune cell content between different subgroups.

### 2.8. Development of the Risk Score

We used the “sva” package ComBat function to merge and standardize the gene expression profiles of the TCGA and METABRIC datasets to analyze the relationship between PIDG subgroup-related genes and prognosis. Univariate Cox regression analysis was performed on the intersection of differentially expressed genes (DEGs) between the two datasets. Subsequently, the combined matrix was randomly divided into training and test sets at a 1:1 ratio. In the training set, the expression profiles of genes significantly associated with prognosis were normalized, with this gene expression matrix as the independent variable and survival time and survival status as the response variables, undergoing least absolute shrinkage and selection operator (LASSO) regression analysis [20,21]. The optimal penalty parameter λ was determined through 10-fold cross-validation, corresponding to the number of variables involved in the model. The PIDG score was calculated as the sum of the product of the model gene expression levels and regression coefficients. The effectiveness of the PIDG score in predicting the survival of TNBC patients in the training and test sets was evaluated through Kaplan–Meier survival analysis and receiver operating characteristic (ROC) curves.

### 2.9. Establishment of a Predictive Nomogram

The PIDG score was combined with clinicopathological variables to create a nomogram for TNBC patients. The accuracy and effectiveness of the nomogram were evaluated using calibration curves, ROC curves, and decision curve analysis (DCA) at 1, 3, and 5 years.

### 2.10. Immune Landscape Analysis of PIDG Score

The ESTIMATE algorithm was employed to calculate the immune score, stromal score, and ESTIMATE score for TNBC patients [22]. We compared the difference in tumor microenvironment scores between the high-risk and low-risk groups. The CIBERSORT algorithm was used to assess the relative abundance of 22 immune cell types in TNBC patients [23]. Spearman correlation analysis was performed to explore the relationship between the PIDG score and the immune cell content. Using the Wilcoxon rank-sum test, PIDG score was employed to compare the expression level difference of immune checkpoint genes between the high-risk and low-risk groups.

We evaluated the Tumor Immune Dysfunction and Exclusion (TIDE) score and immune response of TNBC samples using the TIDE database “http://tide.dfci.harvard.edu (accessed on 7 October 2023)” [24]. We compared the difference in immune escape and immune response rates between the high-risk and low-risk groups using the chi-square test and Wilcoxon rank-sum test. Immunophenoscore (IPS) was obtained from The Cancer Immunome Atlas database “https://tcia.at/home (accessed on 8 October 2023)” [25], and the difference in IPS between different PIDG score risk groups was compared.

### 2.11. Mutation and Drug Sensitivity Analysis

The somatic mutation of high- and low-risk TNBC patients was analyzed using the “maftools” R package, and the mutation landscape of these groups was visualized. We utilized the “calcPhenotype” function from the “oncoPredict” package to predict the sensitivity of 198 commonly used chemotherapeutic drugs for TNBC samples [26]. We compared the difference in chemotherapeutic drug sensitivity between the high-risk and low-risk groups using the Wilcoxon rank-sum test.

### 2.12. Statistical Analysis

All statistical analyses were performed using R software version 4.3.1. Wilcoxon tests were used for pairwise group comparisons. Kaplan–Meier survival analysis and log-rank tests were used to compare survival difference between the two groups. Univariate Cox analysis was used to select prognostically significant genes, and LASSO regression analysis was employed to build the prognostic model. ROC curves were used to assess the predictive value of the regression model. A significance level of *p* < 0.05 was considered statistically significant.

## 3. Results

### 3.1. Identification and Molecular Characterization of Primary Immunodeficiency Features in TNBC

**Figure 1:**
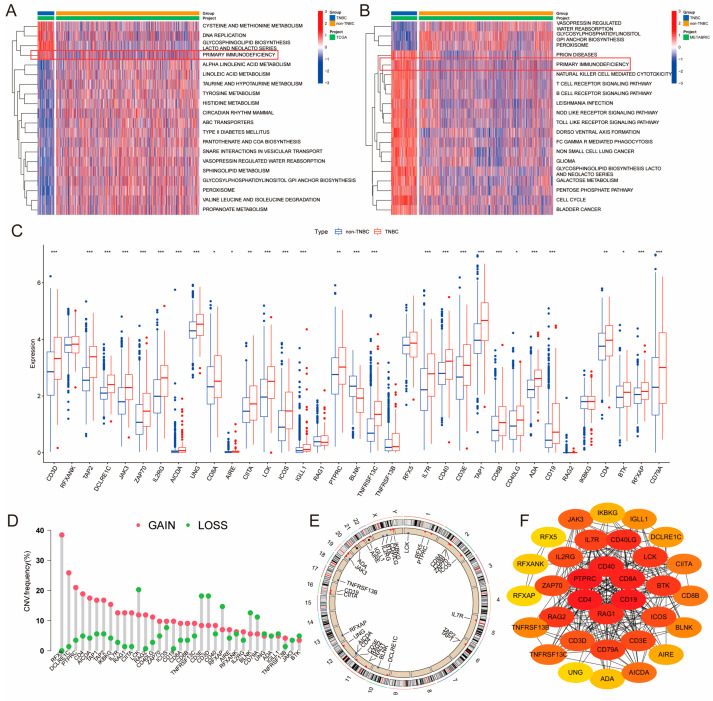
Molecular characterization of PID in TNBC. (**A**,**B**) GSVA heatmaps of TNBC and non-TNBC patients (A-TCGA; TNBC, *n* = 116; non-TNBC, *n* = 997) (B-METABRIC; TNBC, *n* = 320; non-TNBC, *n* = 2189). (**C**) Significance analysis of PIDGs between TNBC and non-TNBC patients. (*, *p* < 0.05; **, *p* < 0.01; ***, *p* < 0.001). (**D**) Copy number variation (CNV) of PIDGs in TNBC patients. (**E**) Chromosomal locations of CNVs for PIDGs in TNBC patients. (**F**) Protein–protein interaction networks (PPI) analysis of PIDGs in TNBC.

To explore the dysregulated functional gene sets in TNBC, we conducted GSVA analysis on samples from TCGA and METABRIC databases, comparing TNBC with non-TNBC samples. The results revealed that the PID pathway was significantly upregulated in TNBC samples in both datasets (Figure 1A,B). The PID pathway comprises 35 genes (Primary Immunodeficiency Genes, PIDGs) that are crucial for the development of various cancers. To further investigate the molecular characteristics of PIDGs, we analyzed their expression levels in the TCGA database. We demonstrated that most PIDGs were significantly overexpressed in TNBC samples compared with non-TNBC samples (Figure 1C).

Subsequently, we analyzed the mutation status of PIDGs, showing that 11.22% of TNBC patients exhibited mutations in PIDGs, with the highest mutation frequencies observed in genes such as *CD4*, *PTPRC*, and *IL2RG* (Figure S1A). The most common genes with copy number increases were *RFX5*, *DCLRE1C*, and *PTPRC*. In contrast, genes such as *LCK*, *CD3E*, and *CD3D* had the highest frequency of copy number decreases (Figure 1D). Copy number variations were predominantly found on chromosomes 10–12 and the X chromosome (Figure 1E).

To investigate the interactions between PIDGs, we conducted a PPI analysis. We found that while all the 35 PIDGs demonstrated significant interactions, the hub genes within PIDGs were *CD4*, *PTPRC*, *CD40*, *CD8A*, *CD19*, and *RAG1A* (Figure 1F). Network of correlation analysis revealed a synergistic interaction among PIDGs (Figure S1B). Kaplan–Meier survival analysis showed that high expression of *ADA*, *AIRE*, *BTK*, *CD40*, *CD4* and *PTPRC* was associated with adverse outcomes in TNBC patients (Figure S1C). In contrast, low expression of *DCLRE1C*, *RFXANK*, *TAP2*, and *TNFRSF13C* correlated with poorer prognosis (Figure S1D). These findings suggest that PIDGs exhibit dysregulated expression in TNBC, manifesting extensive genomic instability that influences patient survival and can be used as a potential molecular target for the clinical treatment of TNBC.

### 3.2. Identification and Comparison of PID Subtypes and Their Biological Features

**Figure 2:**
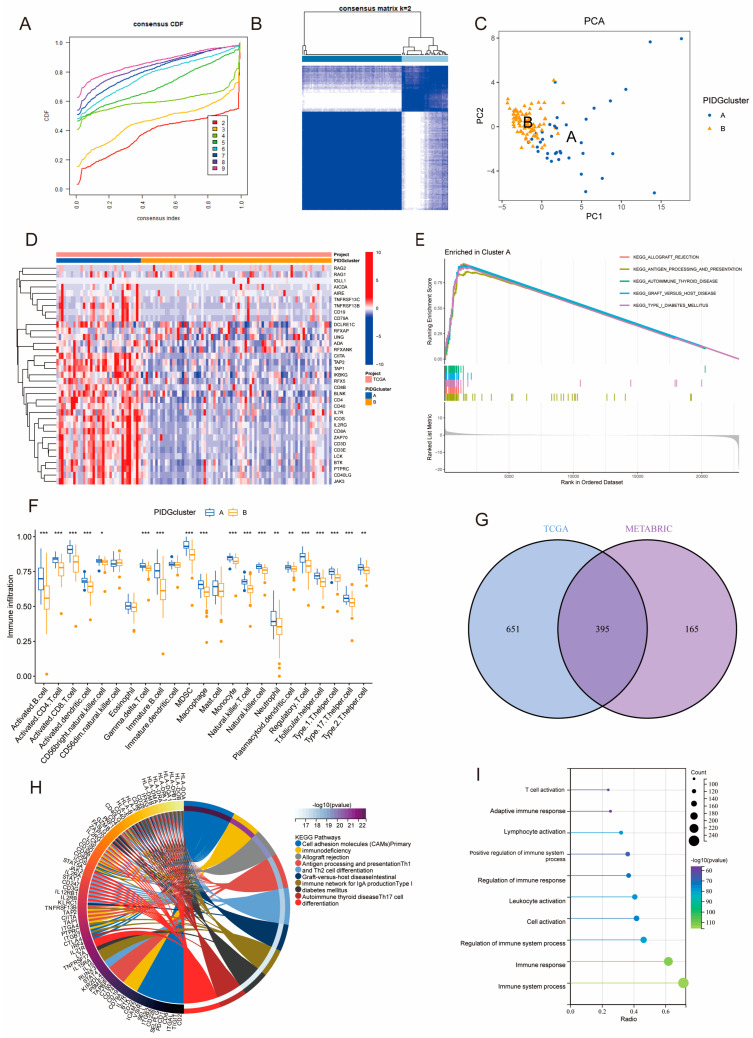
Molecular subtyping based on PIDGs features. (**A**–**F**) Consensus clustering results of TNBC patients in TCGA. (**A**) Cumulative distribution function plot of consensus clustering. (**B**) Heatmap of consensus clustering for k = 2; (**C**) PCA showing the distribution of PIDGs subgroups. (**D**) Expression level distribution of 35 PIDGs among different subgroups. (**E**) GSEA shows molecular functional differences among PIDGs subgroups. (**F**) Differences in immune cell content among different PIDGs subgroups (*, *p* < 0.05; **, *p* < 0.01; ***, *p* < 0.001). (**G**) Venn diagram of the significance analysis of PID clustering in TCGA and METABRIC cohorts. (**H**) KEGG enrichment analysis of differentially expressed genes. (**I**) GO enrichment analysis of differentially expressed genes.

To further investigate the expression patterns of PIDGs in TNBC patients, we performed consensus clustering analysis on the TCGA and METABRIC sample cohorts separately. Based on the gene expression matrix of PIDGs, both cohorts displayed the most stable clustering results at k = 2 (Figure 2A,B and Figure S2A,B). PCA analysis revealed significant inter-group distribution difference between clusters A and B (Figure 2C and Figure S2C), suggesting that the consensus clustering analysis distinguished the cohorts into two groups. Furthermore, the results of differential expression analysis showed that most PIDGs had higher expression levels in cluster A (Figure 2D and Figure S2D).

We conducted GSEA analysis on different PIDGs subgroups in TNBC patients to explore their biological functions. The results showed that patients in cluster A exhibited significant enrichment in immune-related pathways, including allograft rejection, antigen processing and presentation, autoimmune thyroid disease, graft versus host disease, and type I diabetes mellitus (Figure 2E and Figure S2E). Analysis of immune cell infiltration in the microenvironment revealed that, compared with cluster B, cluster A had higher levels of most immune cell types, including activated B cells, activated CD4^+^ T cells, and activated CD8^+^ T cells (Figure 2F and Figure S2F). These results suggest that the subgroup with high expression of PIDGs may have higher immune cell content and more robust immune functions. 

To delve deeper into the biological functions of PIDG subgroups, we examined the entire genome for difference between the subgroups (log|FC| > 1, *p* < 0.05). In both the TCGA and METABRIC databases, we identified 1046 and 560 DEGs, respectively. Notably, 395 genes were found to be differentially expressed in both databases (Figure 2G). Subsequent KEGG and GO analyses of these 395 DEGs revealed significant enrichment in immune-related pathways and functions, including cell adhesion molecules (CAMs), antigen processing and presentation, Th1 and Th2 cell differentiation, Th17 cell differentiation, as well as immune system process, immune response, regulation of immune response, and cell activation (Figure 2H,I). These findings further indicate that PID subtype-associated genes are involved in multiple immune-related biological functions and pathways, highlighting their potential importance in immunotherapy for TNBC.

### 3.3. Construction and Validation of a Prognostic Model Based on the PIDG Score

**Figure 3:**
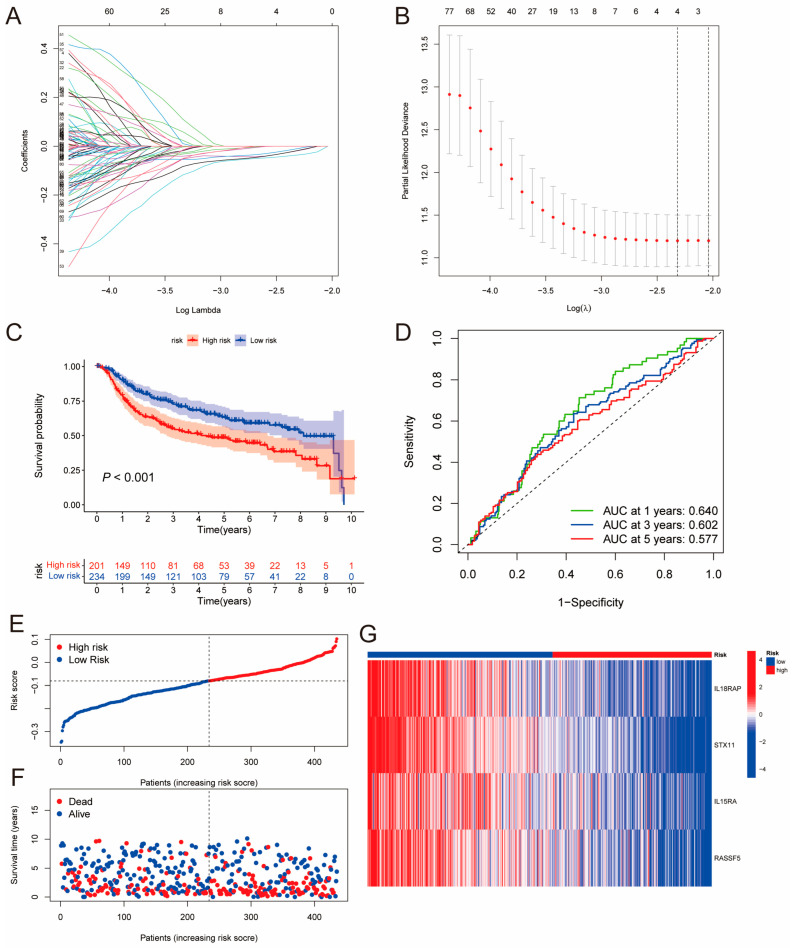
Construction of the PIDG-score-based prognostic model. (**A**) LASSO regression coefficient distribution of differentially expressed genes regulated by PIDGs, each color line represents a gene. (**B**) Selection of the best parameter (lambda) in LASSO regression; (**C**) Kaplan–Meier analysis of the prognosis difference between high- and low-risk groups based on PIDG score. (**D**) ROC curve evaluating the accuracy of the PIDG score prognostic model. (**E**) Ranked dot plot showing the distribution of PIDG score risk scores. (**F**) Scatter plots displaying the survival status distribution of different PIDG score risk scores. (**G**) Heatmap showing the expression of genes involved in constructing the PIDG score model.

PIDGs and their associated genes may play a crucial role in immune regulation. Therefore, we established a prognostic model based on the PIDG score to explore the potential value of these genes in the prognosis analysis of TNBC patients. Firstly, we conducted univariate Cox regression analysis of the influence of 395 DEGs on TNBC patient survival (from the TCGA and METABRIC cohorts, totaling 435 cases). The results revealed that a total of 183 genes were closely associated with the prognosis of TNBC patients (Supplementary Table S1). Subsequently, using a random method, TNBC patients were divided into a training group (218 cases) and a test group (217 cases). LASSO regression analysis was performed on these 183 prognosis-related genes in the training group. When the mean square error was minimized, the optimal λ was determined, resulting in four variables: *IL18RAP*, *STX11*, *IL15RA*, and *RASSF5* (Figure 3A,B). According to the results of the LASSO regression analysis, the formula for calculating the PIDG score is as follows:

**PIDG score = (−0.0193) × *IL18RAP* + (−0.0364) × *STX11* + (−0.0106) × *IL15RA* + (−0.0187) × *RASSF5***

Then, we calculated the PIDG score for each TNBC sample and classified patients into high-risk and low-risk groups based on the median risk value in the training group. The Kaplan–Meier survival analysis results for the training set, test set, and complete set demonstrated that the overall survival (OS) rate of the low PIDG score group was significantly higher than that of the high PIDG score group (Figure 3C and Figure S3A,B). The area under the ROC curve (AUC) values for the one-, three-, and five-year ROC curves of the complete group were 0.640, 0.602, and 0.577, respectively, indicating that the model had high prognostic predictive ability (Figure 3D). The training and test sets also exhibited similar predictive performance (Figure S3A,B). The risk plot of the PIDG score indicated that with an increase in the PIDG score, the OS of the patients decreased, and the mortality rate increased (Figure 3E,F). Additionally, we observed that the model genes *IL18RAP*, *STX11*, *IL15RA*, and *RASSF5* were relatively highly expressed in the low PIDG score group (Figure 3G). To validate the superiority of PIDG score in prediction, we randomly selected five established TNBC risk scores published within the last three years for evaluation. Compared to other risk scores, the PIDG score demonstrates better performance (Supplementary Table S2 and Figure S3C–G). This series of analyses and results suggest that the PIDG score prognostic model can effectively predict the survival status of TNBC patients.

### 3.4. Construction of a Nomogram by Combining PIDG Score and Clinicopathological Variables

**Figure 4:**
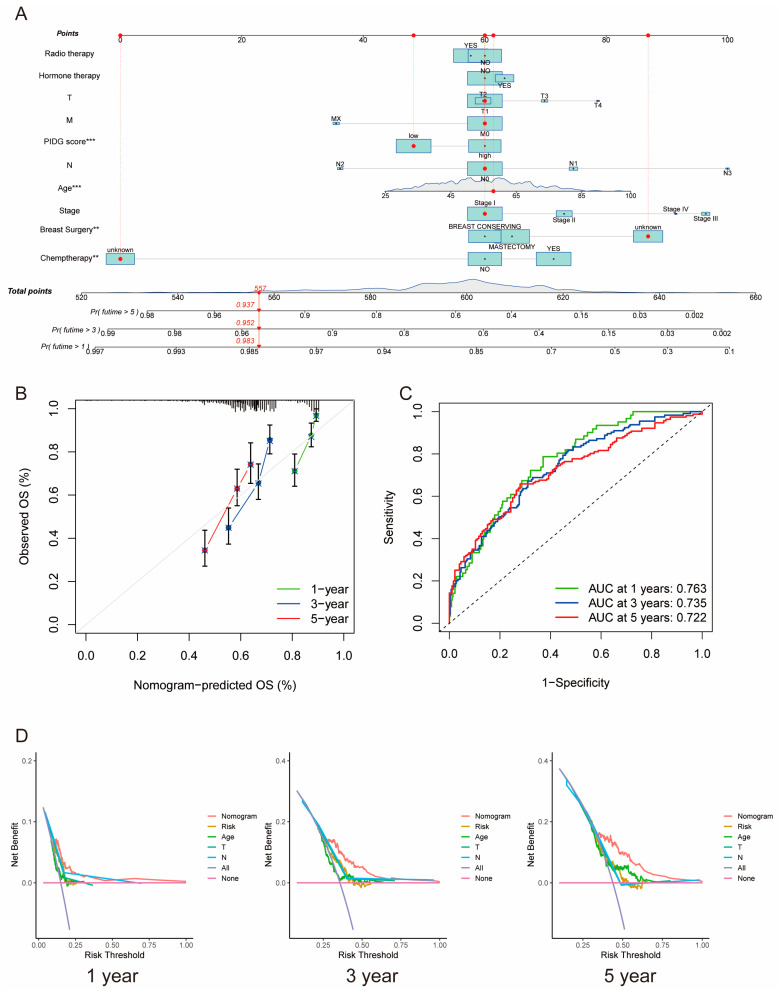
Construction of the clinical nomogram based on PIDG score. (**A**) Nomogram construction based on PIDG score in combination with clinicopathological factors. (**B**) Prediction of one-, three-, and five-year calibration plots; (**C**) ROC curve. (**D**) Decision curve analysis at one, three, and five years to evaluate the model performance. (**, *p* < 0.01; ***, *p* < 0.001).

We utilized PIDG score along with clinicopathological variables such as patient age, chemotherapy, hormone therapy, radiotherapy, breast surgery, staging, tumor size (T), lymph node status (N), and distant metastasis status (M) to construct a nomogram for predicting one-, three-, and five-year OS. PIDG score, patient age, and breast surgery were identified as independent prognostic factors for TNBC (Figure 4A).

Calibration curves demonstrated high consistency between predicted and observed values for one-, three-, and five-year survival rates, indicating the nomogram’s predictive accuracy (Figure 4B). The AUC values for one, three, and five years were 0.763, 0.735, and 0.722, respectively, suggesting excellent prognostic predictive capability of the nomogram (Figure 4C). Moreover, the one-, three-, and five-year DCA curves of the nomogram notably deviated from the reference line, indicating significant net benefits in clinical application (Figure 4D). These results emphasize the critical role of PIDG score in the nomogram and underscore the essential clinical applicability of our nomogram for predicting survival rates in TNBC patients.

### 3.5. Differential Mutation Landscapes between the High- and Low-Risk Patient Groups

**Figure 5:**
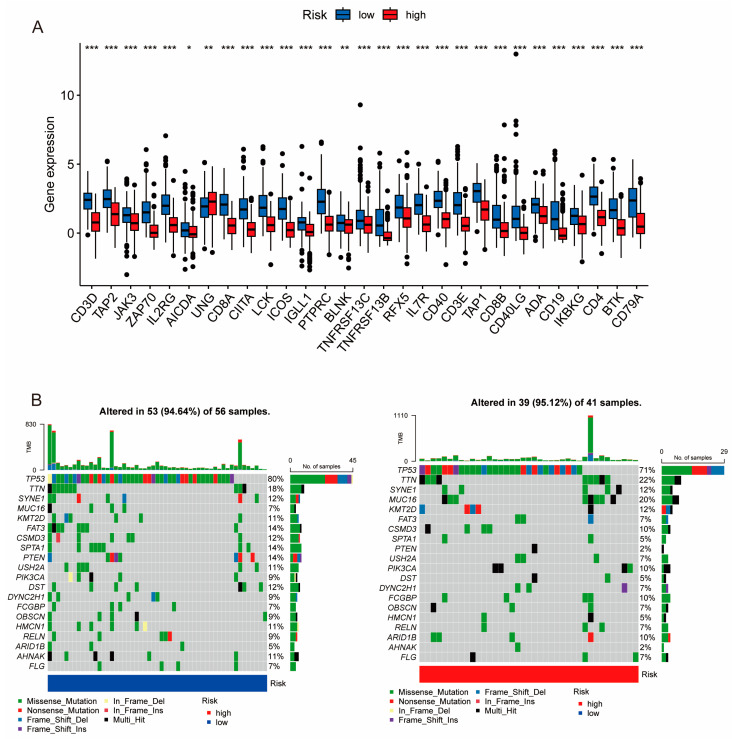
Expression levels of PIDGs and mutation landscape in different PIDG score groups. (**A**) Analysis of PIDGs expression levels in high- and low-risk groups based on PIDG score. (**B**) Mutation landscape in high- and low-risk groups based on PIDG score. (*, *p* < 0.05; **, *p* < 0.01; ***, *p* < 0.001).

To better understand the biological features concerning the PIDG score in TNBC patients, we further analyzed the gene expression patterns in patients with different PIDG scores. A box plot was used to visualize the differential expression of PIDGs between patients with high and low PIDG scores. The results indicated that most PIDGs were significantly upregulated in the low PIDG score group (Figure 5A).

According to the waterfall plot of the high and low PIDG score groups, we observed that among TNBC patients, the five genes with the highest mutation frequencies were *TP53*, *TTN*, *SYNE1*, *MUC16*, and *KMT2D*. Except for *TP53* and *SYNE1*, the mutation frequencies of the other three genes were higher in the high PIDG score group than in the low PIDG score group. Additionally, the somatic mutation rate was slightly higher in the high PIDG score group compared with the low PIDG score group (95.12% vs. 94.64%) (Figure 5B). These results highlight the difference in gene expression and mutation patterns among patients with different PIDG scores.

### 3.6. Relationship between PIDG Score and Methylation Modifications

**Figure 6:**
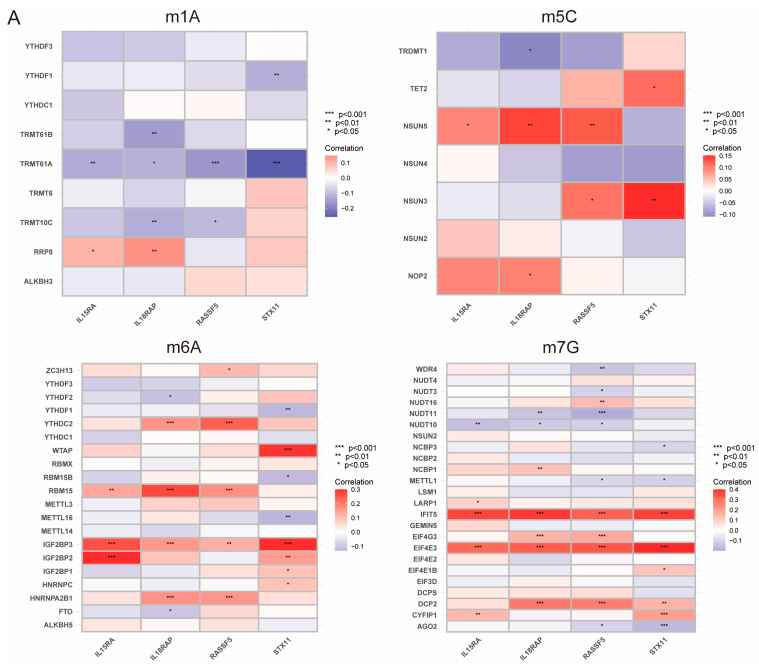

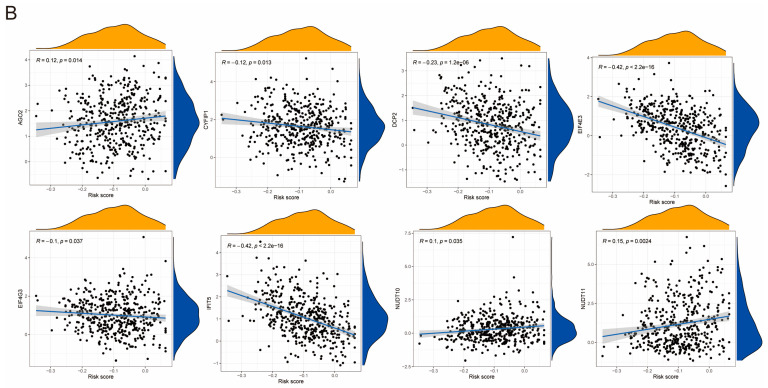
Relationship Between PIDG score and Methylation Modifications. (**A**) Correlation of m1A, m5C, m6A, m7G with modeling genes (*, *p* < 0.05; **, *p* < 0.01; ***, *p* < 0.001). (**B**) Correlation of PIDG score with m7G genes.

RNA modification is an emerging mechanism in gene regulation. This reversible post-transcriptional modification is regulated by “writers” (methyltransferases), “readers”, and “erasers” (demethylases), influencing various molecular functions [27,28,29]. Common RNA modifications include m6A, m5C, m1A, and m7G [30,31,32]. Recent advances in epi transcriptomics have revealed the functional associations between RNA modification and various human diseases [33], and studies have suggested that RNA modification may be improperly regulated in human cancers, making it a potentially ideal target for cancer therapy [34,35,36]. To better understand the regulatory mechanism of PIDG score concerning various methylation levels, we collected the genes related to m1A, m5C, m6A, and m7G modifications and performed correlation analyses with the model genes. We found the highest correlation with m7G (Figure 6A). Additionally, we observed that the PIDG score were positively correlated with *AGO2*, *NUDT10*, and *NUDT11* in m7G modification while negatively correlated with *CYFIP1*, *DCP2*, *EIF4E3*, *EIF4G3*, and *IFIT5* (Figure 6B). We also identified some correlations with other methylation modifications, such as the positive correlation of the PIDG score with *TRMTG1A* and *YTHDF1* in m1A modification (Figure S4A), the negative correlation of PIDG score with *NSUN3* in m5C modification (Figure S4B). In the case of m6A modification, PIDG scores were positively correlated with *YTHDF1* while significantly negatively correlated with *IGFBP2*, *HNRNPA2B1*, *YTHDC2*, *WTAP*, *RBM15*, and *IGF2BP3* (Figure S4C).

While m7G methylation appears to have the highest probability of regulating the PIDG score, other methylation modifications such as m6A may also play a role in the regulation of PIDG score, providing important clues for our understanding of the mechanism of PIDG score in TNBC.

### 3.7. Varying Immune Landscapes between the High- and Low-Risk Patient Groups

**Figure 7:**
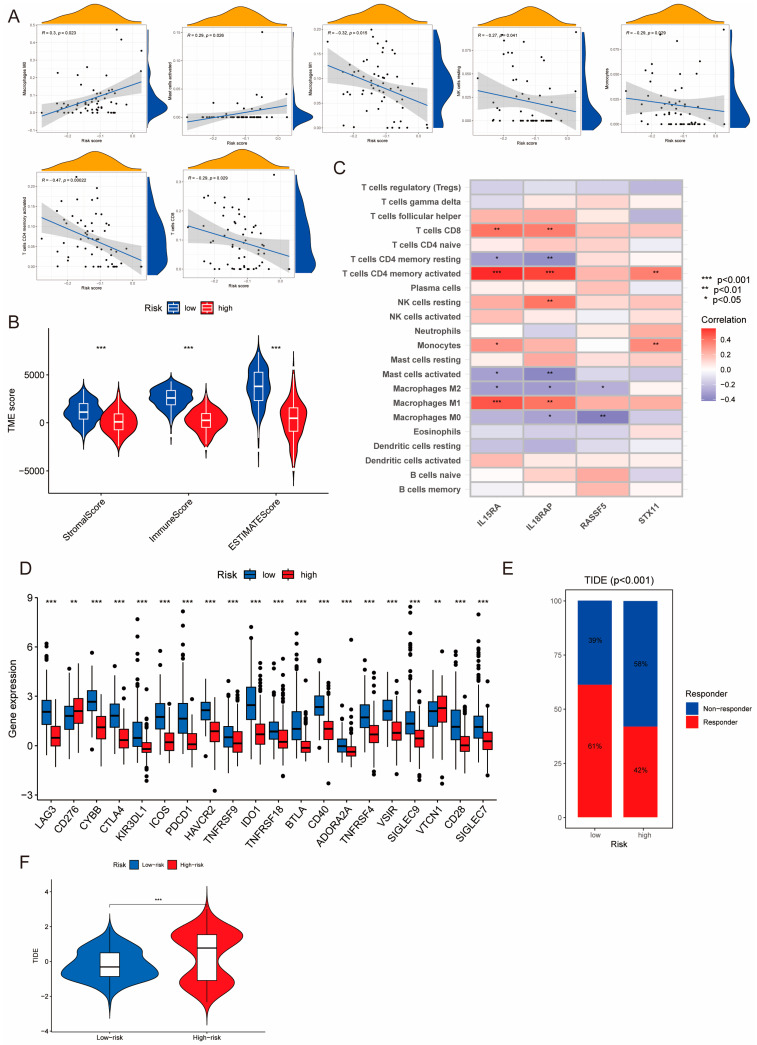
Correlation of PIDG score model with immune function. (**A**) Correlation of PIDG score with immune cell infiltration in the microenvironment. (**B**) Stromal score, immune score, ESTIMATE score (***, *p* < 0.001). (**C**) Correlation of modeling genes of PIDG score with immune cell infiltration in the microenvironment; (*, *p* < 0.05; **, *p* < 0.01; ***, *p* < 0.001). (**D**) Expression levels of immune checkpoint genes in high- and low-PIDG score groups. (**, *p* < 0.01; ***, *p* < 0.001). (**E**) Immune response status in high- and low-risk groups based on PIDG score. (**F**) Immune escape status in high- and low-risk groups based on PIDG score. (***, *p* < 0.001).

TNBC patients with different PIDG scores demonstrated varying immune landscapes. Correlation analysis revealed the positive correlation of PIDG score with Macrophages M0 and activated Mast cells. At the same time, it exhibited s negative correlation with Macrophages M1, Monocytes, NK cells resting, T cells CD4 memory activated, and T cells CD8 (Figure 7A). Further analysis indicated that compared with the high PIDG score group, TNBC patients in the low PIDG score group exhibited higher immune scores, stromal scores, and ESTIMATE scores, potentially aiding in the resistance against tumor growth and dissemination (Figure 7B).

We also found that the model genes *IL15RA* and *IL18RAP* exhibited higher significant correlation with immune cells. Their expression levels showed significant positive correlation with CD8^+^ T cells, CD4^+^ T cells memory activated, and Macrophages M1 while demonstrating significant negative correlation with CD4^+^ T cells memory resting, activated Mast cells, and Macrophages M2 (Figure 7C). There was a substantial difference in the expression of immune checkpoint genes between different PIDG score groups. Specifically, the expression levels of immune checkpoint genes *CTLA4* and *PDCD1* (the gene coding for PD-1) were significantly increased in the low PIDG score group, suggesting an active immune state and a stronger anti-tumor immune response. Conversely, in the high PIDG score group, the immune checkpoint genes *CD276* and *VTCN1* exhibited higher expression levels, possibly associated with immune suppression and warranting more targeted immunotherapy strategies (Figure 7D).

Notably, in terms of immune response, patients in the low PIDG score group demonstrated a higher rate of immune response occurrence compared with the high PIDG score group (61% vs. 42%) (Figure 7E). Additionally, the TIDE score in the high PIDG score group was significantly higher than that in the low PIDG score group (Figure 7F). These findings underscore the distinct immune landscapes in TNBC patients with different PIDG scores, providing valuable insights for the development of personalized immunotherapy.

### 3.8. IPS Scores and Drug Sensitivity Analysis

**Figure 8:**
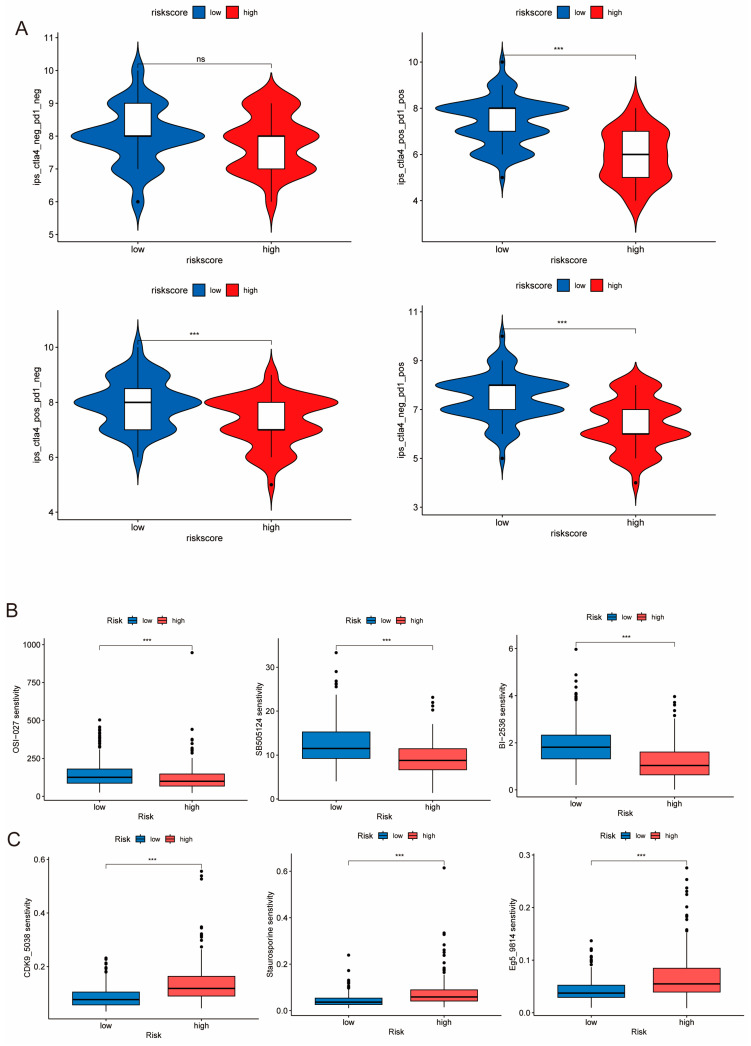
Sensitivity of different PIDG score groups to drug treatments. (**A**) IPS score analysis of the efficacy of immune checkpoint inhibitor treatment. (ns, non-significant; ***, *p* < 0.001) (**B**) Comparison of sensitivity to commonly used chemotherapeutic drugs. (***, *p* < 0.001). (**C**) Drugs exhibited higher sensitivity in the low PIDG score group. (***, *p* < 0.001).

Immunotherapy is currently a promising treatment strategy in TNBC [13]. To investigate the response levels to immunotherapy in patients with different PIDG scores, we compared the Immunophenotype Scores (IPS) between the different groups. The results showed that the low PIDG score group exhibited higher IPS scores (Figure 8A), indicating that patients with low PIDG scores may be more sensitive to immunotherapy. Through the assessment and comparison of the sensitivity to commonly used breast cancer chemotherapeutic or targeted drugs, we also found that the low PIDG score group demonstrated greater sensitivity to drugs such as cisplatin, cyclophosphamide, docetaxel, epirubicin, olaparib, and paclitaxel (Figure S5).

Additionally, we identified that certain drugs, including OSI-027, sb505124, and BI2536, exhibited enhanced sensitivity in the high PIDG score group (Figure 8B). In contrast, drugs such as Eg5_9814, CDK9_5038, and Staurosporine demonstrated improved sensitivity in the low PIDG score group (Figure 8C). These findings illustrate the difference in immunotherapy response and drug sensitivity among patients with different PIDG scores, providing vital information for developing personalized treatment strategies.

## 4. Discussion

PID can affect various components of the immune system, and patients with PID are prone to infections and exhibit manifestations of autoimmune diseases, malignancies, or other immune dysregulations [7]. It has been suggested that when DNA repair proteins are genetically defective, lymphocyte development may be impaired, leading to PID in patients and compromising tumor immune surveillance. Patients with PID often tend to have stronger predispositions to cancer development [37]. While lymphomas account for the majority, an increasing incidence of non-lymphoma tumors, such as breast cancer, has also been observed [38].

Through GSVA, we identified aberrant expression of primary immunodeficiency in TNBC patients. Compared with non-TNBC samples, the PID pathway showed a significant upregulation trend. A considerable proportion of patients had mutations in PIDGs, suggesting that primary immunodeficiency may play an important role in the occurrence and development of TNBC. Expressly, PPI network analysis and survival analysis indicated that genes such as *CD4*, *PTPRC*, *CD40*, *CD8A*, *CD19*, and *RAG1A* play a significant role in PIDGs and are closely related with the survival rate of TNBC patients. These findings provide important clues for further exploration of the role of immunodeficiency-related pathways in TNBC.

Next, through integrated analysis of multi-omics data from the TCGA and METABRIC databases, we divided TNBC patients into high-risk and low-risk subtypes based on the molecular expression patterns of PIDGs. The two subgroups showed significant differences in immune infiltration and function. The expression difference of PIDGs may be the driving factor in our search for appropriate treatment strategies. We further analyzed the common DEGs related to the characteristics of the two subgroups in the TCGA and METABRIC cohorts and performed KEGG and GO analyses, with most of these differentially expressed genes enriched in immune-related functions or pathways. These findings highlight the importance of immune-related therapies in TNBC treatment. Although immune checkpoint-based immunotherapy has been investigated at different clinical stages for TNBC [39], the PIDG score provides new direction for investigation of the applicability of immunotherapy in TNBC patients for improved outcomes.

Furthermore, we investigated the molecular regulatory mechanisms of innate immunodeficiency in TNBC patients. We successfully constructed a LASSO regression model and screened out four model genes related to innate immunodeficiency, namely *IL18RAP*, *STX11*, *IL15RA*, and *RASSF5*. Subsequently, we created a nomogram and found that the PIDG score model accurately predicts the prognosis of patients. Another perspective revealed that, apart from surgical treatment, other treatment modalities do not significantly improve the prognosis of TNBC patients. Compared with other risk scores, we observed better performance in PIDG score. This might be attributed to our approach in constructing the risk model and the number of samples selected. We utilized a total of 435 cases for validating and constructing the PIDG score, whereas the sample size in other risk scores was comparatively smaller than ours.

RNA methylation is one of the most prevalent forms of RNA modification within cells, and modified RNA may be secreted extracellularly, serving as a molecular target in clinical liquid biopsies of patient blood and other fluids. While there are numerous modifications that occur on DNA, detecting DNA modifications through liquid biopsies is challenging. Hence, the authors of this paper consider analyzing the impact of primary immunodeficiency on target genes from the standpoint of RNA modifications, aiming to provide theoretical support for future clinical applications.

We found that PIDG score is likely primarily regulated by m7G methylation. This does not mean that the role of other methylation modifications is ignored. Our research has revealed that m5C methylation is mainly associated with the writer genes, while m6A methylation is more related to the reader genes. These findings suggest that in the regulation of PIDG score, m6A methylation may be more involved in the recognition and regulation processes of genes, while m5C methylation may predominantly influence gene coding and transcription. This difference provides crucial clues for a deeper understanding of the regulatory mechanism of PIDG score.

We found that PIDG score patients have different immune microenvironments and mutation landscapes, which may serve as prognostic biomarkers and provide patient stratification for the selection of different immunotherapy for TNBC.

There are certain limitations regarding the standardized treatment approach for TNBC, such as paclitaxel or anthracycline drugs. This treatment regimen is effective for less than 30% of TNBC patients and the recurrence and mortality rates remain higher than non-TNBC subtypes [40]. Based on the findings of patients with different PIDG scores, immunotherapy is more effective for patients with a low PIDG score. Additionally, our analysis of the sensitivity of some commonly used chemotherapeutic drugs revealed that most drugs are effective only for TNBC patients with a low PIDG score, with poor efficacy for those with a high PIDG score. Encouragingly, we have identified some drugs, such as OSI-027 (mTOR inhibitor), sb505124 (TGFβR inhibitor), and BI2536 (PLK-1 inhibitor), which demonstrate higher sensitivity in patients with a high PIDG score. These drugs have been validated in vitro experiments for their inhibitory effects on TNBC cell growth and metastasis [41,42,43], offering new treatment prospects for patients with a high PIDG score. Additionally, we have discovered some drugs targeting low PIDG score that have not been used in clinical treatment, such as Eg5_9814, CDK9_5038, and Staurosporine, which may provide new options for clinical treatment.

## 5. Conclusions

In summary, this study comprehensively elucidates the important role of PID in the development of TNBC. A prognostic model and clinical prediction tool based on PIDG offers novel scientific evidence for the clinical treatment and prognosis assessment of TNBC patients. Our research provides a new theoretical basis for developing more effective personalized immunotherapy strategies and drug selection, offering new hope for the prognosis and survival of TNBC patients.

## Figures and Tables

**Table 1:** RNA methylation-related genes of m6A/m5C/m1A/m7G.

RNA Methylation	Writer	Reader	Eraser
m6A	*METTL3*, *METTL14*, *METTL16*, *WTAP*, *KIAA1499*, *RBM15*, *RBM15B*, *RBM1*, *ZC3H13*	*YTHDC1*, *YTHDC2*, *YTHDF1*, *YTHDF2*, *YTHDF3*, *IGF2BP1*, *IGF2BP2*, *IGF2BP3*, *HNRNPA2B1*, *HNRNPC*, *HNRNPG*, *RBMX*, *FMR1*, *LRPPRC*	*FTO*, *ALKBH5*
m5C	*NOP2*, *NSUN1*, *NSUN2*, *NSUN3*, *NSUN4*, *NSUN5*, *NSUN7*, *TRDMT1*,	*ALYREF*	*TET2*, *YBX1*
m1A	*TRMT6*, *TRMT61A*, *TRMT61B*, *TRMT61C*, *TRMT10C*, *BMT2*, *RRP8*	*YTHDF1*, *YTHDF2*, *YTHDF3*, *YTHDC1*	*ALKBH1*, *ALKBH3*
m7G	*METTL1*, *WDR4*, *NSUN2*	*AGO2*, *CYFIP1*, *EIF4E*, *EIF4E1B*, *EIF4E2*, *EIF4E3*, *GEMIN5*, *LARP1*, *NCBP1*, *NCBP2*, *NCBP3*, *EIF3D*, *EIF4A1*, *EIF4G3*, *IFIT5*, *LSM1*, *NCBP2L*, *SNUPN*	*DCP2*, *DCPS*, *NUDT10*, *NUDT11*, *NUDT16*, *NUDT3*, *NUDT4*, *NUDT4B*

## Data Availability

Data are contained within the article and supplementary materials.

## Supplementary Materials

The following supporting information can be downloaded at: https://www.mdpi.com/article/10.3390/genes14122172/s1, Figure S1: Supplement for PIDGs Genomic Characterization; Figure S2: Characterization of PIDG Subgroups in TNBC from the METABRIC; Figure S3: Comparison of Different Prognostic Models; Figure S4: Correlation Analysis of PIDG Score with RNA Modifications; Figure S5: Analysis of Chemotherapeutic Drug Sensitivity in PIDG Scores; Table S1: Univariate Cox Output; Table S2: Comparison of Prognostic Model Performance in TNBC-related Studies.

## References

[1] Jiang Y.Z., Ma D., Suo C., Shi J., Xue M., Hu X., Xiao Y., Yu K.D., Liu Y.R., Yu Y. (2019). Genomic and Transcriptomic Landscape of Triple-Negative Breast Cancers: Subtypes and Treatment Strategies. Cancer Cell.

[2] Garrido-Castro A.C., Lin N.U., Polyak K. (2019). Insights into Molecular Classifications of Triple-Negative Breast Cancer: Improving Patient Selection for Treatment. Cancer Discov..

[3] Denkert C., Liedtke C., Tutt A., von Minckwitz G. (2017). Molecular alterations in triple-negative breast cancer-the road to new treatment strategies. Lancet.

[4] Thaventhiran J.E.D., Lango Allen H., Burren O.S., Rae W., Greene D., Staples E., Zhang Z., Farmery J.H.R., Simeoni I., Rivers E. (2020). Whole-genome sequencing of a sporadic primary immunodeficiency cohort. Nature.

[5] Kersey J.H., Spector B.D., Good R.A. (1975). Primary immunodeficiency and malignancy. Birth Defects Orig. Artic. Ser..

[6] Baleydier F., Bernard F., Ansari M. (2020). The Possibilities of Immunotherapy for Children with Primary Immunodeficiencies Associated with Cancers. Biomolecules.

[7] Riaz I.B., Faridi W., Patnaik M.M., Abraham R.S. (2019). A Systematic Review on Predisposition to Lymphoid (B and T cell) Neoplasias in Patients with Primary Immunodeficiencies and Immune Dysregulatory Disorders (Inborn Errors of Immunity). Front. Immunol..

[8] von Minckwitz G., Martin M. (2012). Neoadjuvant treatments for triple-negative breast cancer (TNBC). Ann. Oncol. Off. J. Eur. Soc. Med. Oncol..

[9] Leon-Ferre R.A., Goetz M.P. (2023). Advances in systemic therapies for triple negative breast cancer. BMJ Clin. Res. Ed..

[10] Jia H., Truica C.I., Wang B., Wang Y., Ren X., Harvey H.A., Song J., Yang J.M. (2017). Immunotherapy for triple-negative breast cancer: Existing challenges and exciting prospects. Drug Resist Updat..

[11] Kudelova E., Smolar M., Holubekova V., Hornakova A., Dvorska D., Lucansky V., Koklesova L., Kudela E., Kubatka P. (2022). Genetic Heterogeneity, Tumor Microenvironment and Immunotherapy in Triple-Negative Breast Cancer. Int. J. Mol. Sci..

[12] Wang J., Ge J., Wang Y., Xiong F., Guo J., Jiang X., Zhang L., Deng X., Gong Z., Zhang S. (2022). EBV miRNAs BART11 and BART17-3p promote immune escape through the enhancer-mediated transcription of PD-L1. Nat. Commun..

[13] Keenan T.E., Tolaney S.M. (2020). Role of Immunotherapy in Triple-Negative Breast Cancer. J. Natl. Compr. Cancer Netw. JNCCN.

[14] Zhou Z., Yu X., Chen Y., Tan X., Liu W., Hua W., Chen L., Zhang W. (2023). Inhibition of the B_7_-H_3_ immune checkpoint limits hepatocellular carcinoma progression by enhancing T lymphocyte-mediated immune cytotoxicity in vitro and in vivo. Clin. Transl. Oncol..

[15] Curtis C., Shah S.P., Chin S.F., Turashvili G., Rueda O.M., Dunning M.J., Speed D., Lynch A.G., Samarajiwa S., Yuan Y. (2012). The genomic and transcriptomic architecture of 2,000 breast tumours reveals novel subgroups. Nature.

[16] Wang E., Li Y., Ming R., Wei J., Du P., Zhou P., Zong S., Xiao H. (2021). The Prognostic Value and Immune Landscapes of a m(6)A/m(5)C/m(1)A-Related LncRNAs Signature in Head and Neck Squamous Cell Carcinoma. Front. Cell Dev. Biol..

[17] Chen J., Song Y.W., Liang G.Z., Zhang Z.J., Wen X.F., Li R.B., Chen Y.L., Pan W.D., He X.W., Hu T. (2022). A Novel m7G-Related Gene Signature Predicts the Prognosis of Colon Cancer. Cancers.

[18] Hänzelmann S., Castelo R., Guinney J. (2013). GSVA: Gene set variation analysis for microarray and RNA-seq data. BMC Bioinform..

[19] Mayakonda A., Lin D.C., Assenov Y., Plass C., Koeffler H.P. (2018). Maftools: Efficient and comprehensive analysis of somatic variants in cancer. Genome Res..

[20] Tibshirani R. (1997). The lasso method for variable selection in the Cox model. Stat. Med..

[21] Friedman J., Hastie T., Tibshirani R. (2010). Regularization Paths for Generalized Linear Models via Coordinate Descent. J. Stat. Softw..

[22] Yoshihara K., Shahmoradgoli M., Martínez E., Vegesna R., Kim H., Torres-Garcia W., Treviño V., Shen H., Laird P.W., Levine D.A. (2013). Inferring tumour purity and stromal and immune cell admixture from expression data. Nat. Commun..

[23] Chen B., Khodadoust M.S., Liu C.L., Newman A.M., Alizadeh A.A. (2018). Profiling Tumor Infiltrating Immune Cells with CIBERSORT. Methods Mol. Biol..

[24] Fu J., Li K., Zhang W., Wan C., Zhang J., Jiang P., Liu X.S. (2020). Large-scale public data reuse to model immunotherapy response and resistance. Genome Med..

[25] Charoentong P., Finotello F., Angelova M., Mayer C., Efremova M., Rieder D., Hackl H., Trajanoski Z. (2017). Pan-cancer Immunogenomic Analyses Reveal Genotype-Immunophenotype Relationships and Predictors of Response to Checkpoint Blockade. Cell Rep..

[26] Maeser D., Gruener R.F., Huang R.S. (2021). oncoPredict: An R package for predicting in vivo or cancer patient drug response and biomarkers from cell line screening data. Brief. Bioinform..

[27] Liu N., Dai Q., Zheng G., He C., Parisien M., Pan T. (2015). N(6)-methyladenosine-dependent RNA structural switches regulate RNA-protein interactions. Nature.

[28] Ma N., Zhu Z., Hu J., Pang J., Yang S., Liu J., Chen J., Tang W., Kuang H., Hu R. (2023). Case report: Detection of fetal trisomy 9 mosaicism by multiple genetic testing methods: Report of two cases. Front. Genet..

[29] Zhang Y., Jiang J., Ma J., Wei Z., Wang Y., Song B., Meng J., Jia G., de Magalhães J.P., Rigden D.J. (2023). DirectRMDB: A database of post-transcriptional RNA modifications unveiled from direct RNA sequencing technology. Nucleic Acids Res..

[30] Ma J., Song B., Wei Z., Huang D., Zhang Y., Su J., de Magalhães J.P., Rigden D.J., Meng J., Chen K. (2022). m^5^C-Atlas: A comprehensive database for decoding and annotating the 5-methylcytosine (m^5^C) epitranscriptome. Nucleic Acids Res..

[31] Liang Z., Ye H., Ma J., Wei Z., Wang Y., Zhang Y., Huang D., Song B., Meng J., Rigden D.J. (2023). m^6^A-Atlas v2.0: Updated resources for unraveling the N^6^-methyladenosine (m^6^A) epitranscriptome among multiple species. Nucleic Acids Res..

[32] Song B., Huang D., Zhang Y., Wei Z., Su J., Pedro de Magalhães J., Rigden D.J., Meng J., Chen K. (2022). m^6^A-TSHub: Unveiling the Context-specific m(6)A Methylation and m^6^A-affecting Mutations in 23 Human Tissues. Genom. Proteom. Bioinform..

[33] Song B., Wang X., Liang Z., Ma J., Huang D., Wang Y., de Magalhães J.P., Rigden D.J., Meng J., Liu G. (2023). RMDisease V2.0: An updated database of genetic variants that affect RNA modifications with disease and trait implication. Nucleic Acids Res..

[34] Barbieri I., Kouzarides T. (2020). Role of RNA modifications in cancer. Nat. Reviews. Cancer.

[35] Zhang Y., Huang D., Wei Z., Chen K. (2022). Primary sequence-assisted prediction of m(6)A RNA methylation sites from Oxford nanopore direct RNA sequencing data. Methods.

[36] Chen K., Picardi E., Han X., Nigita G. (2023). Editorial: RNA modifications and epitranscriptomics, Volume II. Front. Genet..

[37] de Miranda N.F., Björkman A., Pan-Hammarström Q. (2011). DNA repair: The link between primary immunodeficiency and cancer. Ann. N. Y. Acad. Sci..

[38] Salavoura K., Kolialexi A., Tsangaris G., Mavrou A. (2008). Development of cancer in patients with primary immunodeficiencies. Anticancer. Res..

[39] Yi H., Li Y., Tan Y., Fu S., Tang F., Deng X. (2021). Immune Checkpoint Inhibition for Triple-Negative Breast Cancer: Current Landscape and Future Perspectives. Front. Oncol..

[40] Li Y., Zhang H., Merkher Y., Chen L., Liu N., Leonov S., Chen Y. (2022). Recent advances in therapeutic strategies for triple-negative breast cancer. J. Hematol. Oncol..

[41] Li H., Lin J., Wang X., Yao G., Wang L., Zheng H., Yang C., Jia C., Liu A., Bai X. (2012). Targeting of mTORC2 prevents cell migration and promotes apoptosis in breast cancer. Breast Cancer Res. Treat..

[42] Choi S., Yu J., Kim W., Park K.S. (2021). N-cadherin mediates the migration of bone marrow-derived mesenchymal stem cells toward breast tumor cells. Theranostics.

[43] Song C., Lowe V.J., Lee S. (2021). Inhibition of Cdc20 suppresses the metastasis in triple negative breast cancer (TNBC). Breast Cancer.

