# Supplementary material for: Molecular Characterization and Establishment of a Prognostic Model Based on Primary Immunodeficiency Features in Association with RNA Modifications in Triple-Negative Breast Cancer
Source: Genes (Basel). 2023 Dec 2;14(12):2172. doi: 10.3390/genes14122172 (PMC10743198; doi:10.3390/genes14122172)
Supplement: Supplementary file 1 [file genes-14-02172-s001.zip › Supplementary Figure Legends.pdf]

**Supplementary Figure S1 Supplement for PIDGs Genomic Characterization**

(A) Waterfall plot showing PIDGs mutation status. (B) Circos plot showing the correlation of PIDGs. (C) Kaplan-Meier analysis of PIDGs.

**Supplementary Figure S2 Characterization of PIDG Subgroups in TNBC from the METABRIC**

(A-E) Consensus clustering results of TNBC patients in METABRIC. (A) Cumulative distribution function plot of consensus clustering. (B) Heatmap of consensus clustering with k = 2. (C) PCA shows the distribution of PIDG subgroups. (D) Expression level distribution of 35 PIDGs in different subgroups. (E) GSEA shows molecular functional differences in PIDG subgroups. (F) Differences in immune cell content in different PIDG subgroups (*, $P < 0.05$; **, $P < 0.01$; ***, $P < 0.001$).

**Supplementary Figure S3 Comparison of Different Prognostic Models**

(A) Training set ROC curve. (B) Test set ROC curve. (C-G) ROC curve and Kaplan-Meier analysis based on different risk score prognostic models.

**Supplementary Figure S4 Correlation Analysis of PIDG Score with RNA Modifications**

(A) Correlation of PIDG score with m1A genes. (B) Correlation of PIDG score with m5C genes. (C) Correlation of PIDG score with m6A genes.

**Supplementary Figure S5 Analysis of Chemotherapeutic Drug Sensitivity in PIDG Scores**

Sensitivity of clinically used chemotherapeutic drugs in PIDG score high and low expression groups.
