# Supplementary material for: Molecular Characterization and Establishment of a Prognostic Model Based on Primary Immunodeficiency Features in Association with RNA Modifications in Triple-Negative Breast Cancer
Source: Genes (Basel). 2023 Dec 2;14(12):2172. doi: 10.3390/genes14122172 (PMC10743198; doi:10.3390/genes14122172)

Supplementary Figure S1

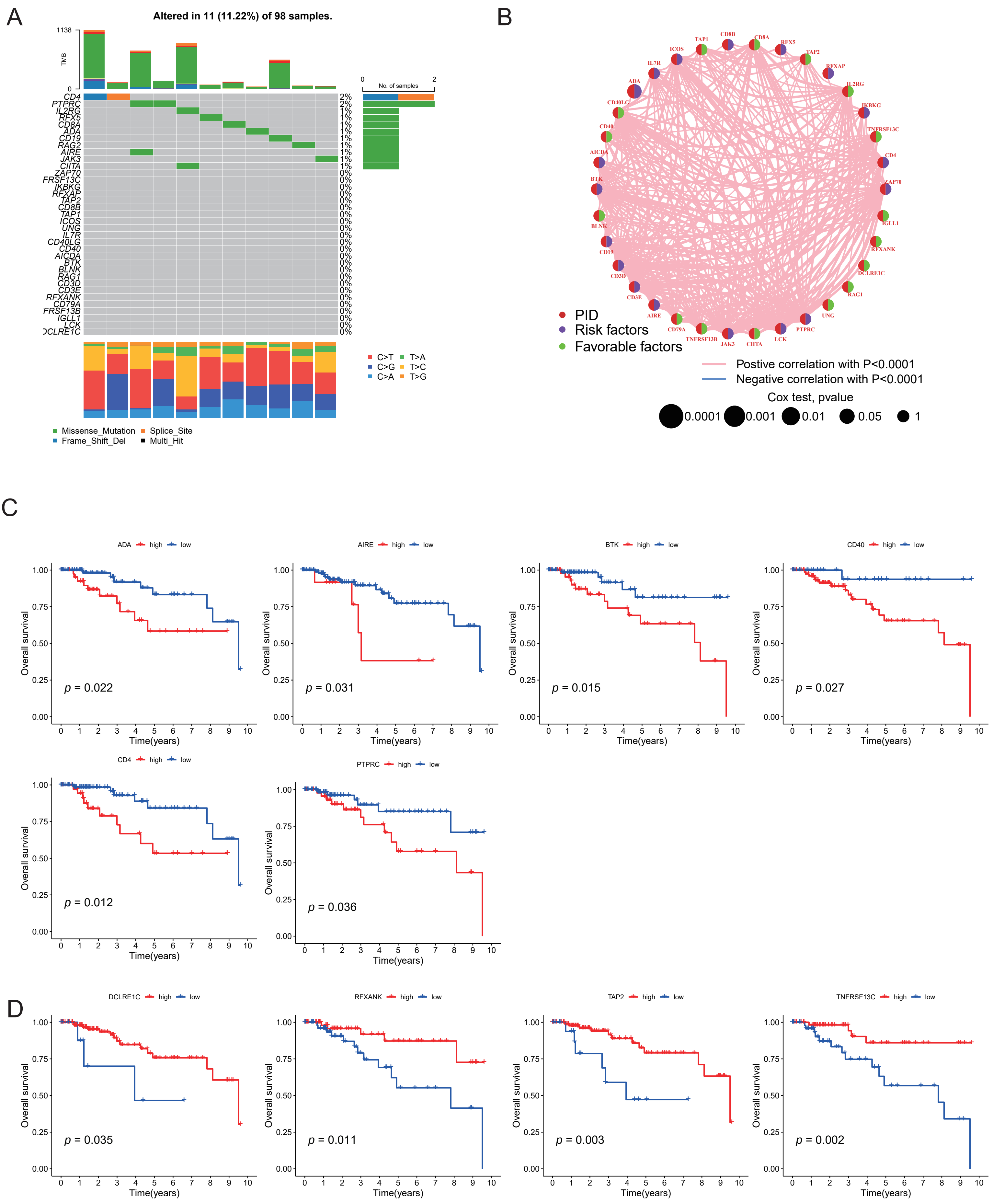

Supplementary Figure S2

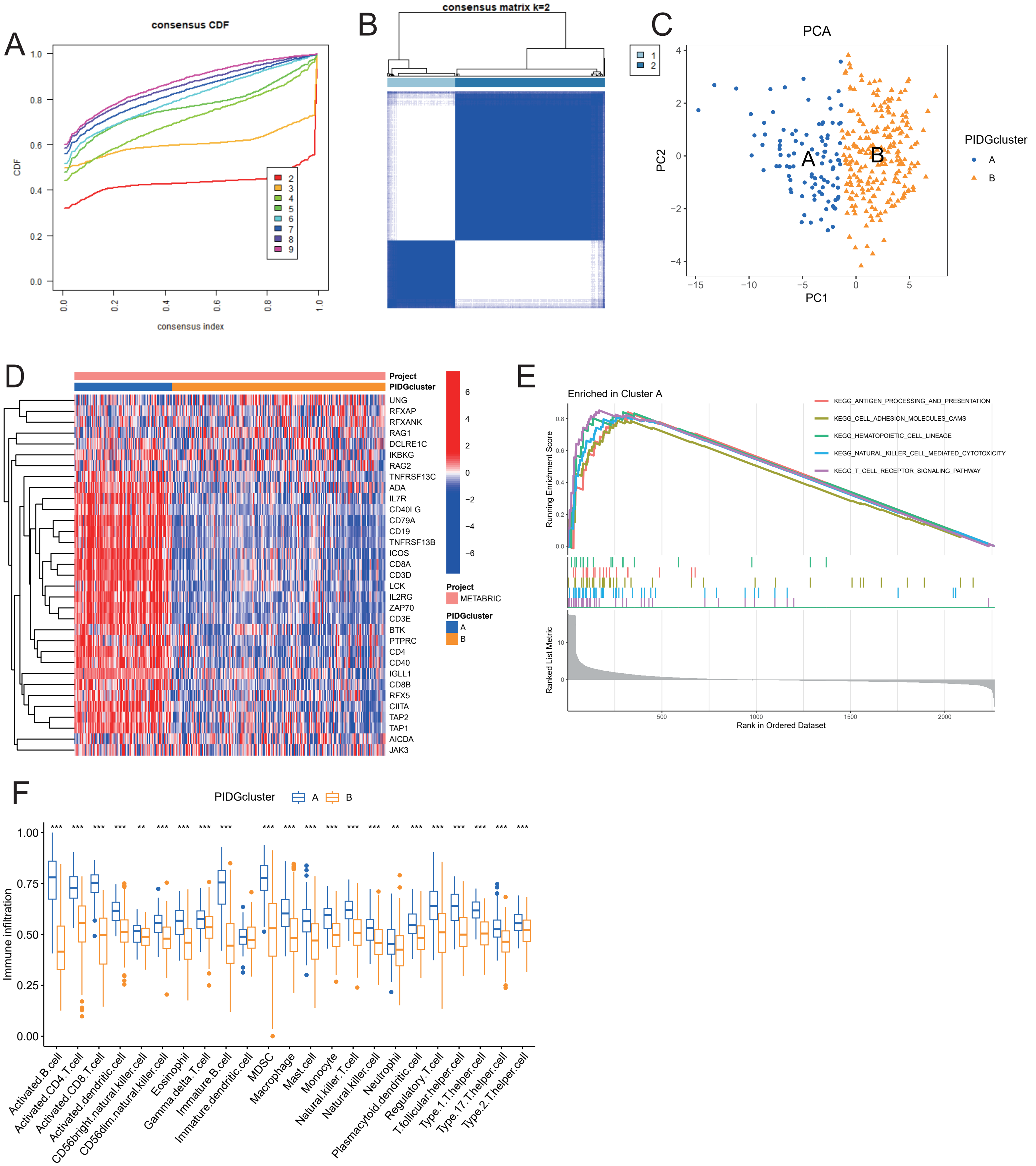

Supplementary Figure S3

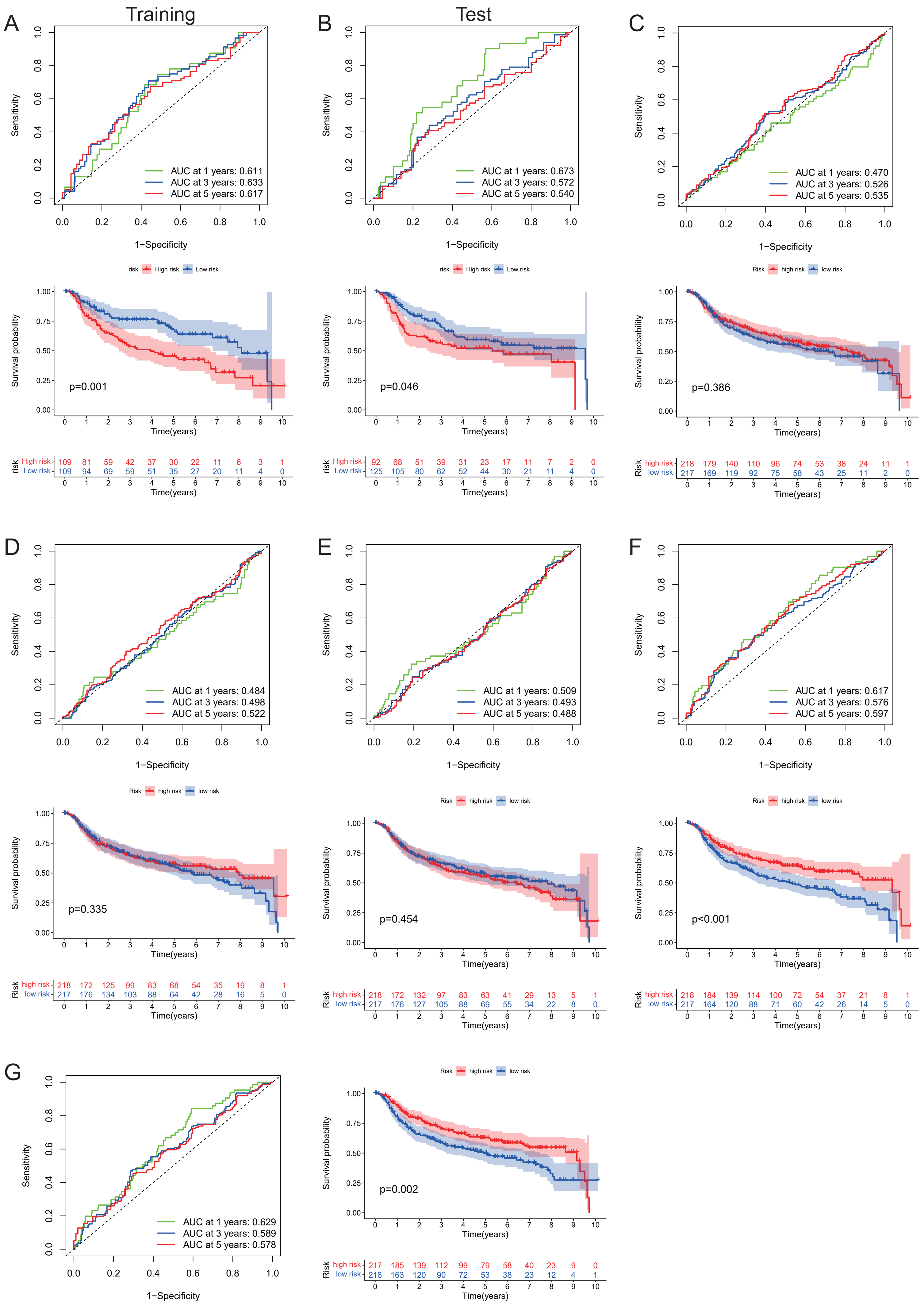

Supplementary Figure S4

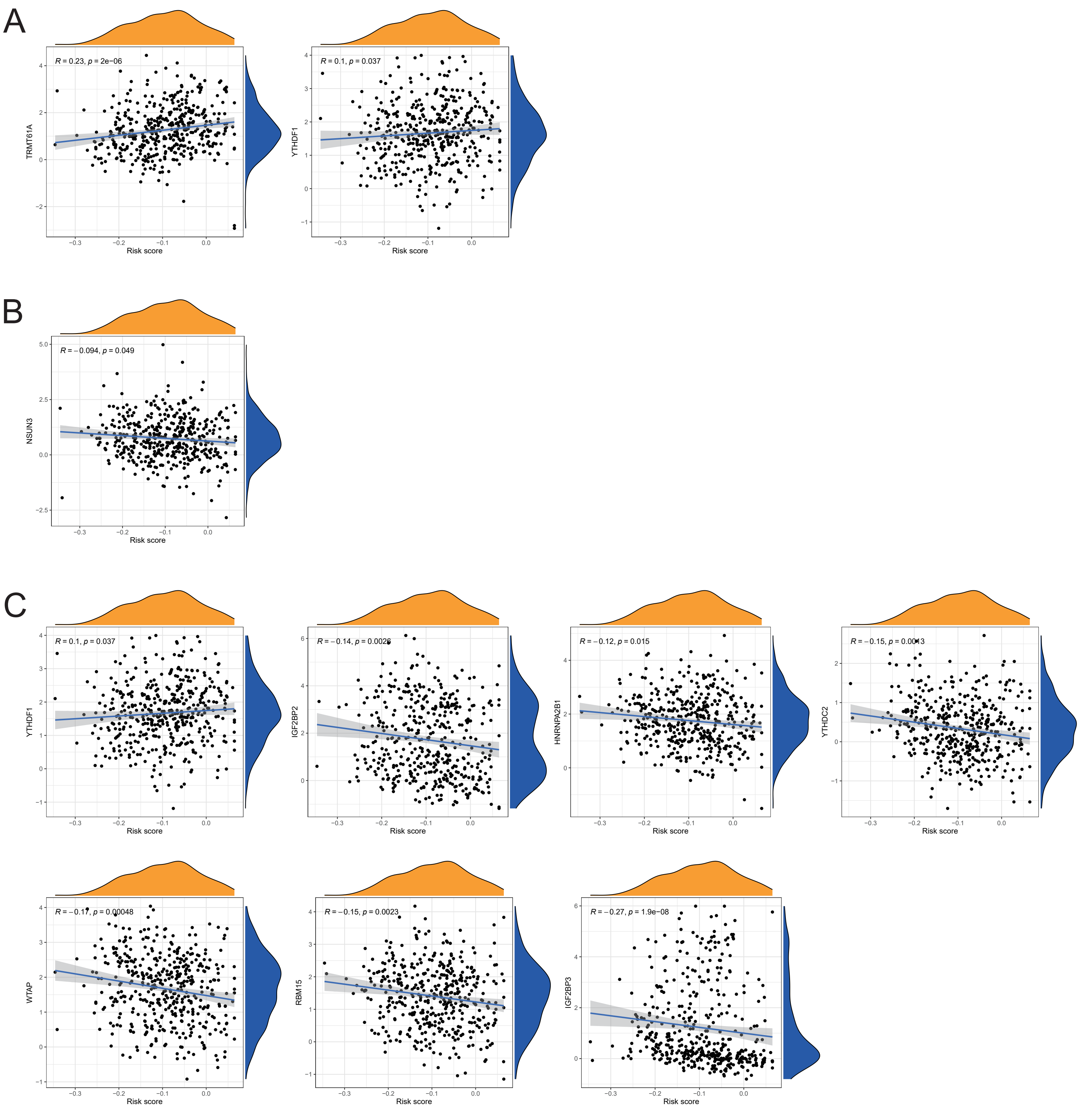

Supplementary Figure S5

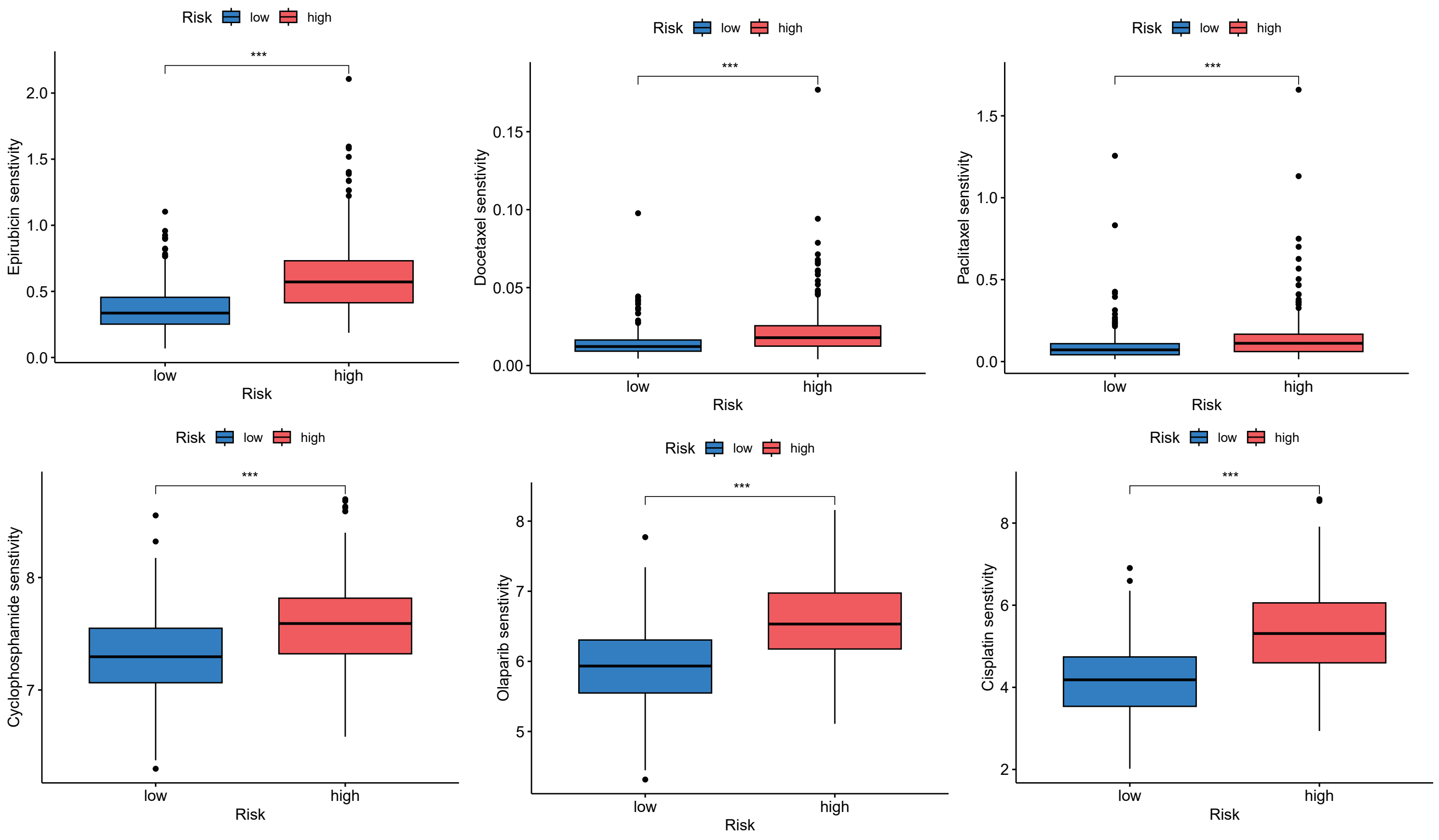

Supplement: Supplementary file 1 [file genes-14-02172-s001.zip › Supplementary Figures_PIDG_Subtypes_TNBC_Characterization_and_Prognostic_Model.pdf]
